# Gadolinium-doped hollow CeO_2_-ZrO_2_ nanoplatform as multifunctional MRI/CT dual-modal imaging agent and drug delivery vehicle

**DOI:** 10.1080/10717544.2018.1428241

**Published:** 2018-01-25

**Authors:** Zuwu Wei, Ming Wu, Zuanfang Li, Zhan Lin, Jinhua Zeng, Haiyan Sun, Xiaolong Liu, Jingfeng Liu, Buhong Li, Yongyi Zeng

**Affiliations:** a Liver Disease Center, the First Affiliated Hospital of Fujian Medical University, Fuzhou, P. R. China;; b The United Innovation of Mengchao Hepatobiliary Technology Key Laboratory of Fujian Province, Mengchao Hepatobiliary Hospital of Fujian Medical University, Fuzhou, P. R. China;; c Academy of Integrative Medicine, Fujian University of Traditional Chinese Medicine, Fuzhou, P. R. China;; d Department of Anesthesiology, Beijing Anzhen Hospital Capital Medical University, Beijing, P. R. China;; e Key Laboratory of OptoElectronic Science and Technology for Medicine of Ministry of Education, Fujian Provincial Key Laboratory for Photonics Technology, Fujian Normal University, Fuzhou, P. R. China

**Keywords:** Drug delivery, nanoplatform, dual-modal imaging, theranostic, multifunctional

## Abstract

Developing multifunctional nanoparticle-based theranostic platform for cancer diagnosis and treatment is highly desirable, however, most of the present theranostic platforms are fabricated via complicated structure/composition design and time-consuming synthesis procedures. Herein, the multifunctional Gd/CeO_2_-ZrO_2_/DOX-PEG nanoplatform with single nano-structure was fabricated through a facile route, which possessed MR/CT dual-model imaging and chemotherapy ability. The nanoplatform not only exhibited well-defined shapes, tunable compositions and narrow size distributions, but also presented a well anti-cancer effect and MR/CT imaging ability. Therefore, the Gd/CeO_2_-ZrO_2_/DOX-PEG nanoplatform could be applied for chemotherapy as well as dual-model MR/CT imaging.

## 1. Introduction

Traditional cancer chemotherapy is often seriously hindered by high-systemic toxicity arising from the lack of tumor specificity when chemotherapeutic drugs are delivered. Currently, nanocarrier-based drug delivery systems (DDS) have emerged as a potential tool to overcome this challenge by either targeted delivery or controlled release of the payload drug in tumors (He & Shi 2014; Kim et al., 2014; Wang et al., 2014; Liu et al., 2015). Moreover, after being encapsulated into nanocarriers, the anticancer drugs will exhibit more favorable pharmacokinetics and tunable bio-distribution which could significantly improve their efficacy (Zhang et al., 2012, 2013; Ma et al., 2014). Despite these, the nano-vehicles can further protect the preserved drugs from destruction by other biomolecules in circulation (Zhang et al., 2015; Banu et al., 2016). In addition to be served as drug carriers, nanoparticles are also used in biomedical imaging such as magnetic resonance imaging (MRI) (Wu et al., 2011; Jiao et al., 2015; Kim et al., 2015; Yang et al., 2015; Ma et al., 2016), computed tomography (CT) (Sun et al., 2015; Li et al., 2016), positron emission computed tomography (PET) (Jin et al., 2010; Sun et al., 2011; Miller et al., 2014; Sun et al., 2015), ultrasound (US) (Zhang et al., 2016), etc. Therefore, theranostics with simultaneously diagnostic and therapeutic capabilities based on nanomaterials, which will provide the detailed information of the tumors, give proper therapy according to the disease conditions, and reflect the treatment efficacy, offer great opportunities in the cure of cancer (Kim et al., 2009; Zhang et al., 2012; Ling et al., 2014; Zhang et al., 2016). However, single imaging modality possesses its intrinsic limitations and weaknesses, which is not competent enough to provide accurate and comprehensive information of tumors, a versatile theranostic agent composed of multimodal imaging ability to guide drug delivery towards tumor for exerting chemotherapeutic role is more applicable (Wang et al., 2016).

Computed tomography (CT) is one of the most reliable imaging modalities that affords high-resolution three-dimensional tomography information of the anatomic structure based on the different X-ray absorption between the normal tissues and the lesions (Betzer et al., 2014; Zhou et al., 2016b). However, the low sensitivity for soft tissue and short imaging times of clinical CT imaging enhancing agents (iodinated compounds) hamper their clinical applications in more detailed tumor imaging. As another powerful noninvasive imaging technique (Izadifar et al., 2016), MRI has been widely used in cancer imaging and cardiovascular disease imaging (Randolph et al., 2016). It offers several advantages including high-spatial resolution, noninvasiveness, high-anatomical contrast, outstanding capacity of differentiating soft tissues. However, unenhanced MRI suffers from low-contrast sensitivity, resulting in the poor discrimination of a tumor from the surrounding tissues. Therefore, it is highly desirable to develop dual-modal imaging agents to combine the CT and MRI, for more accurate disease diagnosis, attributing to the complementary of their individual features.

Recent advances in nanotechnology show that various nanoparticles can be used as contrast agents for MRI/CT dual-modal imaging applications (Chen et al., 2013; Li et al., 2013; Wen et al., 2013; Chen et al., 2015; Chen et al., 2016; Zhou et al., 2016a). For example, He & Shi (2014) reported the synthesis of Au/PPY@Fe_3_O_4_ nanocomposites by decomposing iron on the surfaces of Au NPs in the presence of polypyrrole followed by subsequent functionalization via a surfactant exchange process (Feng et al., 2015). Zhu et al. adopted a heating maturation process to prepare monodisperse Au nanoparticles, and then fabricated a heterostructured Au-Fe_3_O_4_ nanoparticles by thermal degradation method linking of the Fe_3_O_4_ NPs, the finally formed Au-Fe_3_O_4_ nanoparticles have very good colloidal stability (Zhu et al., 2014). Compared with blending various components into one nano-system, versatile nanoparticles integrating all the functionalities within one structure or component is another strategy to fabricate multi-modal contrast agents. For instance, Li’s group reported a fluorine-18-labeled Gd^3+^/Yb^3+^/Er^3+^ co-doped NaYF_4_ nanophosphors, which was used in multimodality PET/MR/UCL imaging (Zhou et al., 2011).

The CeO_2_-ZrO_2_ nanoparticle obtained from pyrolysis with a hollow structure had been widely investigated for high performance de-NOx monolith catalysts (Li et al., 2017). Inspired from their facile route-synthesis, huge interior and high X-ray absorption of Ce/Zr, we expect to utilize this nanoparticle as CT imaging contrast and drug delivery carrier for cancer theranostic. However, these two prospects have not been previously demonstrated and documented neither *in vitro* nor *in vivo*. Moreover, integrating other imaging modality like MRI but without complicating their structure is also very favorable, which can further improve the diagnostic ability of the tumor. Herein, we develop a multifunctional nanoplatform of Gd/CeO_2_-ZrO_2_-PEG for drug delivery and CT/MRI dual-modal imaging, in which the core components of Gd/CeO_2_-ZrO_2_ are integrated *in situ* by one-pot synthesis rather than the complicated post-blending method. Furthermore, the chemotherapeutic drug of DOX was loaded in hollow interior of Gd/CeO_2_-ZrO_2_-PEG. The obtained Gd/CeO_2_-ZrO_2_/DOX-PEG was characterized by transmission electron microscopy (TEM), dynamic light scattering (DLS), Fourier transform infrared spectroscopy (FT-IR). And the magnetic and X-ray attenuation properties of these nanoplatforms were also evaluated in detail. Moreover, the anti-cancer ability of Gd/CeO_2_-ZrO_2_/DOX-PEG was studied *in vitro* and *in vivo*. In addition, we have also investigated the MR and CT imaging Gd/CeO_2_-ZrO_2_/DOX-PEG.

## 2. Experimental

### 2.1 Materials

Ce(NO_3_)_3_·6H_2_O, Gd(NO_3_)_3_·6H_2_O, ZrOCl_2_, ethylene glycol and methoxy polyethylene glycol amine (Mw:2000, mPEG-NH_2_) were purchased from Sigma Aldrich Co., Ltd. Acetone and cyclohexane were obtained from Sinopharm Chemical Reagent Co., Ltd. 1-Octadecene and methanol was purchased from Alladin Company. Cell Counting Kit (CCK-8) was obtained from Dojindo laboratories. Penicillin-streptomycin, fetal bovine serum (FBS), and Dulbecco’s Modified Eagle Medium (DMEM) were purchased from Gibco BRL. All of these materials were used as received without further purification. Deionized water was used throughout the studies.

### 2.2 Synthesis of gadolinium-doped hollow CeO_2_-ZrO_2_ nanoparticles

The synthesis procedure of gadolinium-doped hollow CeO_2_-ZrO_2_ nanoparticles was according to the literature with modifications (Liang et al., 2008). Briefly, 1 mL of 0.5 M Ce(NO_3_)_3_ and 0.25 mL of 0.5 M Gd(NO_3_)_3_ solution were added into 30 mL glycol with stirring, then the mixture was sealed and heated at 180 °C for 16 h. After the reactants cool down to room temperature, 0.5 mL of 0.5 M ZrOCl_2_ solution was added. Then the mixture was sealed and re-heated for another 3h at 180 °C. The gadolinium-doped hollow CeO_2_-ZrO_2_ nanoparticles (Gd/CeO_2_-ZrO_2_) were finally obtained and washed thrice by ethanol with centrifugation at 8000 r/min for 15 min.

### 2.3 Surface PEGylation of Gd/CeO_2_-ZrO_2_

The surface PEGylation process was implemented according to the literature with a certain extent modification. Gd/CeO_2_-ZrO_2_ (0.4 g) and succinic anhydride (0.2 g, 2 mmol) were dissolved in 50 mL of anhydrous CH_2_Cl_2_, DMAP (245 mg, 2 mmol) was then added to the solution, and the mixture was stirred at room temperature for 24 h (Yao et al., 2017). After reaction completion, the Gd/CeO_2_-ZrO_2_-COOH was obtained and washed by ethanol with centrifugation at 8000 r/min for 10 min three times. Then, PEG-NH_2_ (Mn 2000, 0.2 g), Gd/CeO_2_-ZrO_2_-COOH, 1-ethyl-[3-(3-dimethylamino)propyl] carbodiimide hydrochloride and sulfo-N-hydroxysuccinimide were dissolved in anhydrous dimethyl sulfoxide (DMSO, 20 mL) and reacted 24 h at room temperature. The mixture was centrifuged at 10,000 r/min for 15 min and subsequently washed three times with ethanol (Li et al., 2014). The obtained Gd/CeO_2_-ZrO_2_-PEG samples were then re-dispersed in 10 mL of ethanol for future usage.

### 2.4 Doxorubicin loading in Gd/CeO_2_-ZrO_2_-PEG

Firstly, prior to the drug-loading preparation, hydrophobic drug of doxorubicin (DOX) was obtained through treating the doxorubicin hydrochloride (DOX·HCl) with a 2-fold molar amount of triethylamine in DMF for 10 h. Then, 5 mL of doxorubicin (DOX) in THF solution (1 mg/mL) was added into Gd/CeO_2_-ZrO_2_-PEG aqueous solution (10 mL, 4 mg/mL), and then the mixture was stirred at room temperature for 12 h under dark condition. Afterwards, the DOX-loaded particles (Gd/CeO_2_-ZrO_2_/DOX-PEG) were obtained from the mixture by centrifugation at 8000 r/min for 10 min and washed three times with ultrapure water. Finally, the Gd/CeO_2_-ZrO_2_/DOX-PEG sample was re-dispersed in 20 mL of ultrapure water for usage in future, and the DOX content was determined by measuring their absorbance at the wavelength of 480 nm through UV-vis spectra (Chen et al., 2012).

### 2.5 Cell culture

HepG-2 cells were cultured in the regular Dulbecco’s Modified Eagle Medium (DMEM) containing 10% fetal bovine serum and 1% penicillin streptomycin at 37 °C in a humidified atmosphere (5% CO_2_) (Wei et al., 2014).

### 2.6 DOX release studies

Efficient drug release at the desired site is very important for drug delivery systems. The pH of tumor site is much lower than normal condition, because of the generated lactic acid due to hypoxia and acidic intracellular organelles. Therefore, the doxorubicin release profile from Gd/CeO_2_-ZrO_2_/DOX-PEG was assessed by a dialysis method at pH 6.8 and 7.4 for simulate the corresponding physiological environments of acidic cellular endosomes in cancer cells and normal physiological environment. Briefly, the sterilized dialysis bags with molecular-weight cutoff 3500 Dalton were used to carry out the drug release experiments (Nam et al., 2013). Duplicate Gd/CeO_2_-ZrO_2_/DOX-PEG (10 mg) was dispersed into 2 mL PBS at pH 6.8, 7.4, respectively. And then the solutions were put into dialysis bags with 1/3 air gap which sealed with dialysis bag holders. The sealed dialysis bags were put into centrifuge tube and then 10 mL PBS (pH 6.8 and 7.4, respectively) was added. These tubes were shaken at a speed of 220 rpm at 37 °C under a light-sealed condition. At certain time intervals, 5 mL of the medium was withdrawn from exterior solution and replaced with an equal volume of fresh PBS. The amount of released DOX at each time point was determined by its UV-vis absorption spectrum using the corresponding standard calibration curve.

### 2.7 Relaxivity and MRI phantom studies at 7.0 T magnetic field

A series of Gd/CeO_2_-ZrO_2_/DOX-PEG nanoparticles’ aqueous solutions with different Gd concentrations (0, 0.05, 0.11, 0.22, 0.45, and 0.9 mM) were prepared for MRI phantom and relaxivity studies. All experiments were performed on a MRI scanner (7.0 T, Bruker Biospoin GmbH, Germany). The longitudinal relaxation rate (r_1_) was determined from the slope of the plot of 1/T_1_ against the Gd concentration (mM). Images of phantom MRI were analyzed with Kodak Molecular Imaging Software.

### 2.8 X-ray attenuation measurements

Different concentrations of Gd/CeO_2_-ZrO_2_/DOX-PEG (0, 0.3, 0.61, 1.22, 2.45 and 4.9 mg/mL) were dispersed in deionized water in a series of 1.5 mL tubes for phantom test. CT images were acquired using a CT system (MX-16, Philips, Netherlands). Images of phantom CT were analyzed with Kodak Molecular Imaging Software. HU values were measured by the Philips MX-16 CT.

### 2.9 Chemotherapy of Gd/CeO_2_-ZrO_2_/DOX-PEG *in vitro*

To test the cytotoxicity of Gd/CeO_2_-ZrO_2_/DOX-PEG nanoparticles, HepG-2 cells were seeded in a 96-well plate at a density of 1 × 10^4^ cells per well and cultured in 5% CO_2_ at 37 °C for 24 h. Then, free DOX or Gd/CeO_2_-ZrO_2_/DOX-PEG nanoparticles were added to the medium, and the cells were incubated in 5% CO_2_ at 37 °C for 48 h. The concentrations of DOX were adjusted to 0, 0.625, 1.25, 2.5, 3.5, 5 μg/mL, respectively. Cell viability was evaluated using the Cell Counting Kit (CCK-8) assay. In addition, the cytotoxicity of the empty Gd/CeO_2_-ZrO_2_-PEG nanoparticles was also assessed by the same method.

### 2.10 Intracellular uptake of Gd/CeO_2_-ZrO_2_/DOX-PEG and the DOX release behavior

HepG-2 cells were plated on 35 mm glass-bottom Petri dish and allowed to adhere for 24 h. After washed with PBS, the cells were plated in a serum medium containing Gd/CeO_2_-ZrO_2_/DOX-PEG with DOX concentration at 2 μg/mL for 2 h, 4 h, 6 h at 37 °C under 5% CO_2_ atmospheres. At the end, the medium was removed and the cells were washed with PBS for three times. Then the cells were fixed with 4% formaldehyde for 10 min, and the cell nuclei were stained with DAPI (5 μM) for another 10 min. Finally, the cells were imaged by CLSM (Zeiss LSM780) with 543 nm laser excitation for DOX and 405 nm laser excitation for DAPI.

### 2.11 Construction of mouse xenograft subcutaneous models

All experimental protocols involved in animals were approved by Animal Ethics Committee of Mengchao Hepatobiliary Hospital of Fujian Medical University, and all experiments were carried out in accordance with the approved guidelines. Female BALB/c nude mice (4 weeks old) were purchased from Wushi Animal Co., Ltd. All animal experiments were carried out under protocols approved by the Institutional Animal Care and Use Committee. HepG-2 cells (1.0 × 10^7^ cells) were subcutaneously transplanted into BALB/c male nude mice. *In vivo* experiments were conducted when the tumors volume reached a size of 150–200 mm^3^.

### 2.12 Chemotherapy *in vivo*

Each group (*n* = 5) of mice were intravenously injected with 100 μL of PBS, DOX, Gd/CeO_2_-ZrO_2_/DOX-PEG (in terms of DOX∼2.5 mg/kg). The treatment was repeated seven times at a time interval of 3 days. Body weight and tumor volume were recorded every 3 days over a whole period of 22 days. During the period of therapy, the length and width of the tumors were measured by digital caliper every 3 days. The tumor volume was calculated by the formula V = ab^2^/2 (a, the longest dimension; b, the shortest dimension) and expressed as the relative tumor growth rate by normalizing with the initial tumor volume (Tian et al., 2014). All mice were sacrificed at the 22nd day post first treatment. To evaluate anti-cancer effects of the nanoparticles, the tumor was collected and examined by hematoxylin-eosin (H&E) and immunohistochemistry (IHC) staining.

### 2.13 *In vivo* MRI and computed tomography (CT) imaging

Gd/CeO_2_-ZrO_2_/DOX-PEG (2 mg·kg^−1^) were intravenously injected into the xenograft mice. T_1_-weighted images of the mice were obtained on a 7.0 T MRI scanner (Bruker Biospoin GmbH, Germany). The imaging parameters were as follows: fast spin echo, TR 400.0 ms, TE 10.0 ms, and slice thickness 2.0 mm. Meanwhile, *in vivo* computed tomography (CT) imaging was acquired by the Philips MX-16 CT. Images of phantom MRI and CT were analyzed by Kodak Molecular Imaging Software.

## 3. Results and discussion

### 3.1 Synthesis and characterization of Gd/CeO_2_-ZrO_2_/DOX-PEG

**Scheme 1.**
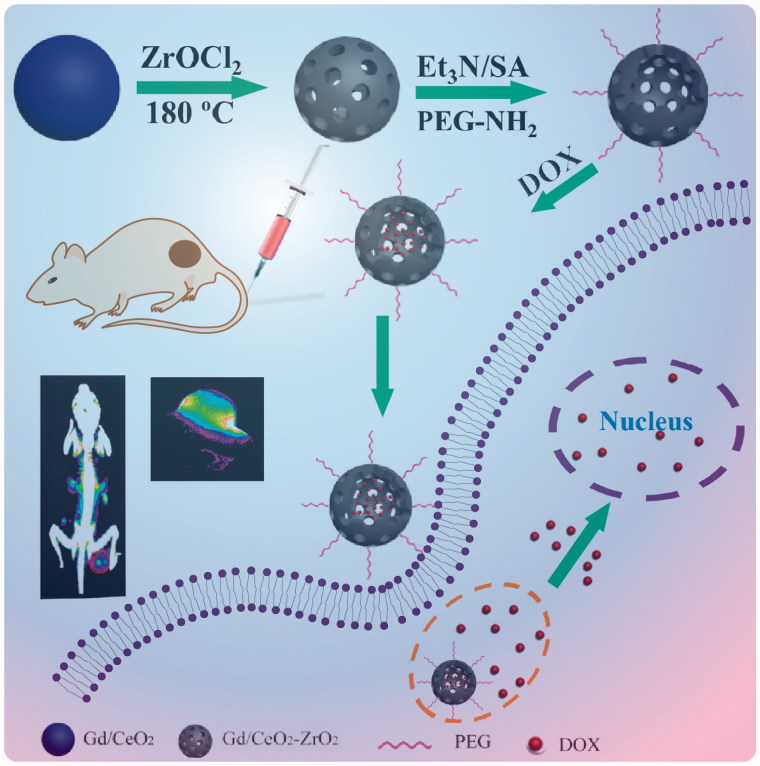
Schematic illustrations of the synthetic procedures for Gd/CeO_2_-ZrO_2_/DOX-PEG and their corresponding application.

**Figure 1.**
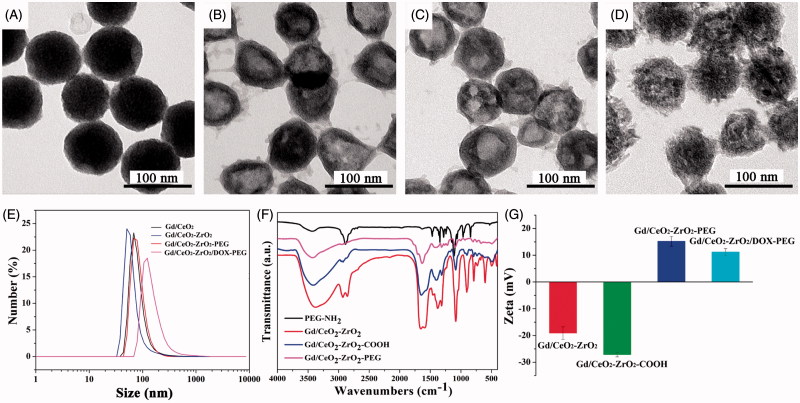
Structure characterizations of Gd/CeO_2_-ZrO_2_/DOX-PEG. TEM image of Gd/CeO_2_ (A), Gd/CeO_2_-ZrO_2_ (B), Gd/CeO_2_-ZrO_2_-PEG (C), Gd/CeO_2_-ZrO_2_/DOX-PEG (D); DLS particle size distribution of Gd/CeO_2_, Gd/CeO_2_-ZrO_2_, Gd/CeO_2_-ZrO_2_-PEG and Gd/CeO_2_-ZrO_2_/DOX-PEG (E); FT-IR spectra of Gd/CeO_2_-ZrO_2_, Gd/CeO_2_-ZrO_2_-PEG and PEG-NH_2_ (F); The zeta potential of Gd/CeO_2_-ZrO_2_, Gd/CeO_2_-ZrO_2_-COOH, Gd/CeO_2_-ZrO_2_-PEG and Gd/CeO_2_-ZrO_2_/DOX-PEG (G).

**Figure 2.**
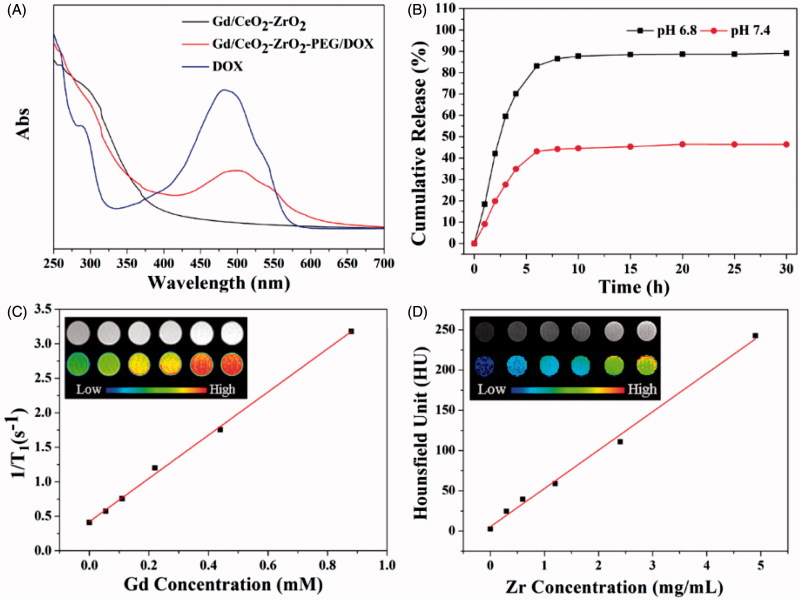
UV-vis spectra of free DOX solution, Gd/CeO_2_-ZrO_2_-PEG and Gd/CeO_2_-ZrO_2_/DOX-PEG (A); *in vitro* release of DOX from Gd/CeO_2_-ZrO_2_-PEG at different pH (B); T_1_-weighted and color-mapped magnetic resonance (MR) images for various Gd^3+^ concentrations and relaxation rate r_1_ (1/T_1_) versus different Gd^3+^ concentrations of Gd/CeO_2_-ZrO_2_/DOX-PEG (inset: T_1_-weighted and color-mapped magnetic resonance (MR) images for various Gd^3+^ concentrations) (C); CT value versus different Zr^4+^ concentrations of Gd/CeO_2_-ZrO_2_/DOX-PEG (inset: CT images and color-mapped images for various Zr^4+^ concentrations) (D).

The synthetic process of Gd/CeO_2_-ZrO_2_/DOX-PEG is illustrated in Scheme 1. Monodispersed Gd-doped CeO_2_ nanospheres have been synthesized via a hydrolysis process in glycol (Figure 1(A)), which exhibit the monodispersity and monosize features with a size of around 85 nm. The Gd/CeO_2_ nanospheres possess high reactivity and diffusing rates which led it to become a good template for the formation of a hollow structure after introducing ZrOCl_2_ via the Kirkendall effect potentially specified as two aspects: (1) Zr^4+^ can readily dope into Gd/CeO_2_ to form the solid solution; (2) the diffusing rate of the Gd/CeO_2_ is obviously much faster than that of the single-crystal nanostructure (Liang et al., 2008). According to the Kirkendall effect, each component has its own diffusing rate in a diffusing environment. Herein, the different diffusing rate is seen in Zr^4+^ and CeO_2_. The solid solution of Ce_1-_*_x_*Zr*_x_*O_2_ type formed as Zr^4+^ doping into ceria because of different diffusing rate (Liang et al., 2008). At the same time, the diffusing rate of the ceria nanospheres is obviously much faster than that of Zr^4+^ (Liang et al., 2008). The obtained Gd/CeO_2_-ZrO_2_ exhibited well-defined shapes, narrow size distributions, and hollow interiors (Figure 1(B)). Furthermore, from the energy dispersive X-ray (EDX) spectrum (see Figure S1), it can be illustrated that the nanoparticles possess Gd, Ce and Zr element. In addition, the surface areas and pore sizes were evaluated. According to the IUPAC classification, the N_2_ adsorption-desorption isotherms of Gd/CeO_2_-ZrO_2_ vehicle (Figure S2) can be classified as type-IV, further confirming the preservation of the mesoporous structure. The BET surface area and total pore volume of Gd/CeO_2_-ZrO_2_ was 436.7 m^2^ g^−1^. The BJH pore size of Gd/CeO_2_-ZrO_2_ was estimated to be 3.8 nm and 13.2 nm. Moreover, the Gd/CeO_2_-ZrO_2_-PEG was obtained by further surface PEGylation. In detail, the Gd/CeO_2_-ZrO_2_ reacted with succinic anhydride and generated Gd/CeO_2_-ZrO_2_-COOH (Figure S3). Then, the Gd/CeO_2_-ZrO_2_-PEG was obtained through coupling reaction between Gd/CeO_2_-ZrO_2_-COOH and PEG-NH_2_. The surface PEGylation nanoparticles still maintain a uniform particle size and good dispersion after surface functionalization (Figure 1(C)). The successful surface modification of PEG was confirmed by Fourier transform infrared spectrum (FT-IR). As shown in Figure 1(F), for the spectrum of Gd/CeO_2_-ZrO_2_, the peak at 3076 cm^−1^ is attributed to the O–H stretching vibration, and peaks at 2930 and 2857 cm^−1^ are assigned to the asymmetric and symmetric stretching vibrations of methylene (CH_2_), respectively. After modification with PEG-NH_2_, the characteristic band at 3425 cm^−1^ appeared, which is attributed to the N–H stretching vibration. In addition, the zeta-potential of Gd/CeO_2_-ZrO_2_ changed form −18.9 mV to −26.8 mV post-reaction with succinic anhydride, which verified that the carboxyl groups were modified on the surface of Gd/CeO_2_-ZrO_2_. However, the zeta-potential further turned to 16.2 mV after the PEG-NH_2_ conjugation (Figure 1(G)). Therefore, the FT-IR and zeta-potential results suggested the successful modification of PEG on Gd/CeO_2_-ZrO_2_ nanoparticles. In addition, we conducted the Dynamic light scattering (DLS) analysis (Figure 1(E)) of Gd/CeO_2_, Gd/CeO_2_-ZrO_2_, Gd/CeO_2_-ZrO_2_-PEG, which showed an average hydrodynamic diameter of 85 ± 10, 95 ± 10, 95 ± 10 and 120 ± 10 nm, respectively. Finally, the loading of DOX in the hollow of the Gd/CeO_2_-ZrO_2_-PEG was confirmed by UV − vis absorbance spectrometry for the appearance of the characteristic absorption bands of DOX (Figure 2(A)) and also the color change of the Gd/CeO_2_-ZrO_2_-PEG solution after centrifugation (Figure S4). Meanwhile, the drug loading can also be observed from TEM image. As shown in Figure 1(D), DOX is mainly enriched in the surface and hollow cavity of nanoparticles. And the loading efficiency of anticancer drugs DOX reached 10.2 wt%.

### 3.2 *In vitro* drug release studies

Controlled drug release is very important for anti-cancer drug delivery platform. The pH of tumor site is lower than normal due to the generated lactic acid by hypoxia and acidic intracellular organelles. The pH-dependent drug release can minimize premature at physiological pH values (pH 7.4) in blood circulation but can be stimulated at the acidic tumor microenvironment or at the acidic cellular organelle after uptake into tumor cells. Therefore, pH-responsive properties are very important for the exploitation of novel nano-drug. Hence, the DOX release profile from Gd/CeO_2_-ZrO_2_-PEG was assessed by a dialysis method at pH 6.8 and 7.4. As shown in Figure 2(B), the DOX release from Gd/CeO_2_-ZrO_2_-PEG was much lower at pH 7.4 (30%) than at pH 6.8 (87%). The main reason for this phenomenon is hydrophobic DOX changing to hydrophilic doxorubicin hydrochloride at lower pH of 6.8.

### 3.3 MRI and CT images *in vitro*

Gd-doped nanoparticles are usually used as MR positive contrast agents, so Gd/CeO_2_-ZrO_2_-PEG nanoplatform is evaluated regarding their T_1_-weighted MR images and relaxivity parameter (r_1_) using an MRI scanner. As the molar concentration of Gd/CeO_2_-ZrO_2_/DOX-PEG ranged from 0 to 0.9 mM, the T_1_-weighted MR images became more brighter (Figure 2(C) inset). To present this dose-dependent contrast enhancement more clearly, colored T_1_-weighted MR images are also provided (Figure 2(C) inset). As the concentration increased, the color of the MR images changed from blue to red, indicating the signal changes from low to high level. Quantitative analysis showed that R_1_ (1/T_1_) value of our nanocomposites was linearly increased along with the Gd concentrations (Figure 2(C)); from the slopes of these curves, the r_1_ values were calculated to be 4.63 s^−1^·mM^−1^. These results suggested that Gd/CeO_2_-ZrO_2_/DOX-PEG could be applied as a T_1_-MRI contrast agent.

To the best of our knowledge, Zr-doped NPs have been reported as a novel CT contrast agent, owing to the high X-ray attenuation effect (Shi et al., 2015; Tan et al., 2016). The CT imaging effect of Gd/CeO_2_-ZrO_2_/DOX-PEG nanoplatform with different concentrations was also evaluated by X-ray CT. The CT signal gradually increased, as the molar concentration of Gd/CeO_2_-ZrO_2_/DOX-PEG increased from 0 to 4.9 mg/mL (Figure 2(D) inset). As plotted in Figure 2(D), the Hounsfield units (HU) values enhanced linearly with the increase of concentration of Gd/CeO_2_-ZrO_2_/DOX-PEG nanoplatform aqueous solution. The slope of the HU value for nanoplatform was about 25.23. Meanwhile, the CT imaging ability of Gd/CeO_2_-ZrO_2_-PEG without drug loading was also evaluated (Figure S5), and the results demonstrated that the loaded drug has no influence on the CT imaging ability. The result indicates that drug loading does not influence the CT imaging effect and Gd/CeO_2_-ZrO_2_/DOX-PEG nanoplatform can be ultilized as an X-ray contrast.

### 3.3 *In vitro* cytotoxicity assessment

**Figure 3.**
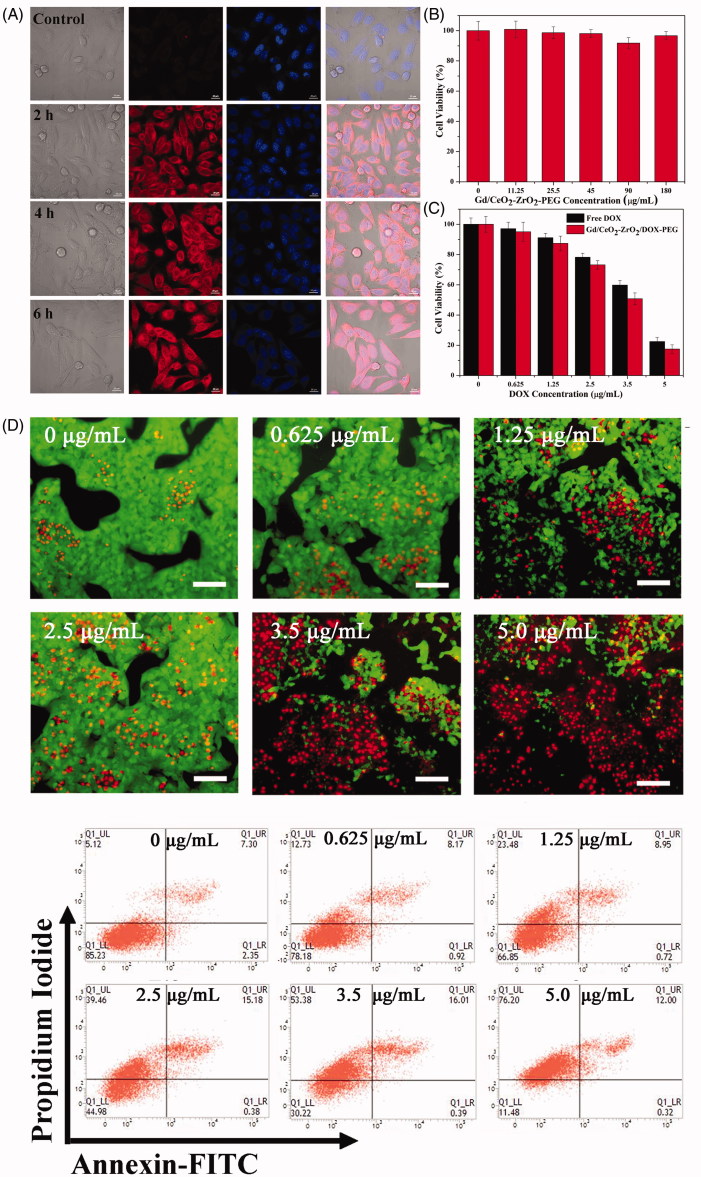
Intracellular uptake Gd/CeO_2_-ZrO_2_/DOX-PEG and DOX release at different incubation time (A); Cell viability of HepG-2 cells treated with different concentration of Gd/CeO_2_-ZrO_2_-PEG (B), Free DOX and Gd/CeO_2_-ZrO_2_/DOX-PEG (C) for 48 h; Fuorescence microscopic images of HepG-2 cells stained by the LIVE/DEAD Cell Vitality Assay Kit with different concentrations of Gd/CeO_2_-ZrO_2_/DOX-PEG for 48 h (D); Flow cytometry analysis of HepG-2 cell apoptosis induced by different concentrations of Gd/CeO_2_-ZrO_2_/DOX-PEG for 48 h as indicated using Annexin V-FITC/PI apoptosis detection assay (E).

Low or none toxicity is a vital criterion of any nanomaterial fabricated for biomedical applications. To explore the application of Gd/CeO_2_-ZrO_2_-PEG nanoparticles as a MRI/CT dual-modal contrast agent and carrier of anti-cancer drug, we first tested their cytotoxicity on HepG-2 cells via the CCK-8 assay. As shown in Figure 3(B), even at the high concentration (180 μg/mL), the viability of cells was still over 95% after incubation for 48 h, suggesting a well biocompatibility of the NPs *in vitro*.

### 3.4 Intracellular uptake of Gd/CeO_2_-ZrO_2_/DOX-PEG and DOX release

To investigate the Gd/CeO_2_-ZrO_2_/DOX-PEG uptake and intracellular DOX release, confocal fluorescence microscopy was conducted to trace DOX distribution in the cells (Figure 3(A)). Incubated with the Gd/CeO_2_-ZrO_2_/DOX-PEG for 2 h, strong red fluorescence arising from DOX is observed in the cell cytoplasm. With the incubation time increased to 4 h, some of the DOX fluorescence could be observed in the cell nuclei. Most significantly, majority of DOX entered into the nuclei, as the prolonging of incubation time to 6 h. Note that the DOX kills cells via its intercalation with DNA backbone, thus, these results demonstrated Gd/CeO_2_-ZrO_2_/DOX-PEG had the potential to kill the cancer cells.

### 3.5 *In vitro* chemotherapy of Gd/CeO_2_-ZrO_2_/DOX-PEG

HepG-2 cells were employed to evaluate the *in vitro* anticancer effect of the Gd/CeO_2_-ZrO_2_/DOX-PEG through CCK-8 assay. As shown in Figure 3(C), a dose-dependent cell viability of Gd/CeO_2_-ZrO_2_/DOX-PEG nanoparticles was found as their concentration ranged from 0 to 5 μg/mL. After 48 h incubation, the cell viability significantly decreased to 22.3%, at the containing DOX concentration of 5 μg/mL. To further intuitively observe the cell killing effect of the Gd/CeO_2_-ZrO_2_/DOX-PEG, HepG-2 cells with our nanoparticles treatment was stained by LIVE/DEAD Cell Vitality Assay Kit, in which the live cells and dead cells could been distinguished though observation of green and red fluorescent cells, respectively. As shown in Figure 3(D), the cells treated with low concentration (0.625 μg/mL) nanoparticles exhibited a very little red fluorescence of dead cells. However, the dead cells with red fluorescence became more obvious, while the number of the live cells with green fluorescence was gradually declined as the nanoparticles concentration increased to 5 μg/mL. Most significantly, nearly all the cells were dead at the containing DOX of 5 μg/mL. This result is consistent with that obtained from the CCK-8 assay. We further investigated whether the cell killing of our Gd/CeO_2_-ZrO_2_/DOX-PEG was through the cell apoptosis pathway, which was determined by flow cytometry using Annexin V-FITC/PI apoptosis detection assay. As shown in Figure 3(E), the majority of cells were localized in the lower left quadrant with more than 85.2% of the viable cells in the control group, indicating no apparent cell death. Compared with control group, the viable cells were decreased after incubation with Gd/CeO_2_-ZrO_2_/DOX-PEG and the dose-dependent cell viability was found. At the DOX concentration of 5 μg/mL, the percentages of survive cells, apoptotic cells and necrosis cells were found to be 11.4%, 12% and 76.2%, respectively. These results clearly suggested that the cell killing of our nanoparticles occurred mainly through the cell necrosis but not apoptosis.

### 3.6 *In vivo* biosafety evaluation

**Figure 4.**
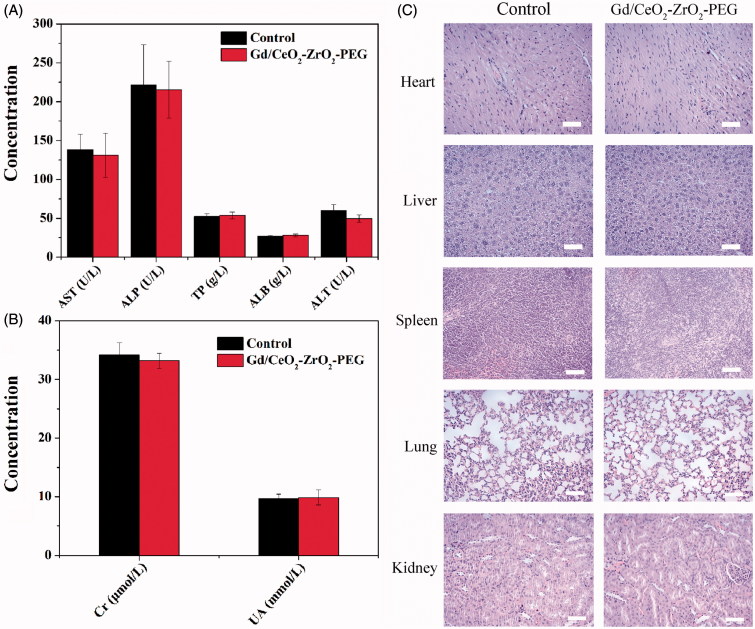
Serum biological parameters of liver (A) and kidney (B) obtained from mice at 7 days after being exposed to Gd/CeO_2_-ZrO_2_-PEG (*n* = 5), or PBS solution (*n* = 5, control); hematoxylin and eosin (H&E)-stained tissue sections from mice at 7 days after injected with Gd/CeO_2_-ZrO_2_-PEG (*n* = 5), or PBS solution (*n* = 5, control). Tissues were harvested from heart, liver, spleen, lung and kidney (C).

Before *in vivo* chemotherapy application of DOX-loaded Gd/CeO_2_-ZrO_2_-PEG, the potential toxicology of free Gd/CeO_2_-ZrO_2_-PEG in female BALB/c mice was carefully studied from the following aspects. Firstly, the blood biochemical indicators were utilized to investigate the influence of Gd/CeO_2_-ZrO_2_-PEG on the liver and kidney function, and the results were shown in Figure 4(A). Representative liver biochemical parameters (AST, ALP, TP, ALB and ALT) in the Gd/CeO_2_-ZrO_2_-PEG-treated group exhibited no statistic difference as compared with the control group injected with saline, suggesting the low hepatotoxicity of Gd/CeO_2_-ZrO_2_-PEG (Wu et al., 2016). Similarly, there was no difference in the UA and Cr level between the Gd/CeO_2_-ZrO_2_-PEG-treated group and the control group, revealing that the Gd/CeO_2_-ZrO_2_-PEG had no impact on renal function (Figure 4(B)) (Wu et al., 2016).

Moreover, to further confirm possible organ damage after nanovehicles injection, histological assessments (H&E staining) of representative organs, including heart, liver, spleen, lung, and kidney, were executed. The results were exhibited in Figure 4(C). During examination, no noticeable abnormality was detected in heart, liver, spleen, lung and kidneys. All the above results suggested that Gd/CeO_2_-ZrO_2_-PEG was tolerable and safe *in vivo*.

### 3.7 *In vivo* tumor inhibition evaluation

**Figure 5.**
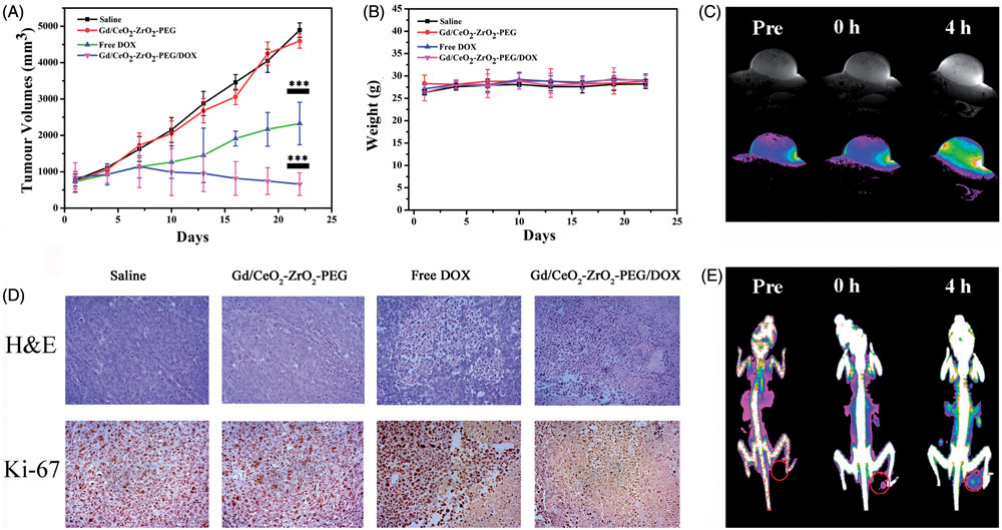
Growth curves of tumors in the mice after chemotherapy treatments (*p < .05, **p < .01, ***p < .001; *n* = 5) (A); relative body weight change of mice in different groups after treatment (*n* = 5) (B); corresponding H&E and Immunohistochemical staining of the tumor tissues after 22 d treatment as indicated (C); T_1_-weighted MR images of tumor acquired at different time intervals post injection of Gd/CeO_2_-ZrO_2_/DOX-PEG (D); CT images of tumor acquired at different time intervals post injection of Gd/CeO_2_-ZrO_2_/DOX-PEG (E).

The anti-cancer activity of Gd/CeO_2_-ZrO_2_/DOX-PEG was assessed using mouse xenograft models with heterotopic, subcutaneous human liver cancers. The free DOX or Gd/CeO_2_-ZrO_2_/DOX-PEG was injected intravenously at DOX concentration of 2.5 mg·kg^−1^ on days 1, 4, 7, 10, 13, 16, 19 and 22, respectively. The results of anti-cancer efficacy were summarized as plots of relative tumor volumes over the course of treatments in Figure 5(A). The PBS and Gd/CeO_2_-ZrO_2_-PEG treated mice showed negligible tumor growth inhibition within 22 days. Mice administrated with free DOX only moderately inhibited tumor growth compared with the PBS treatment. In contrast, the group treated with Gd/CeO_2_-ZrO_2_/DOX-PEG showed continued and effective inhibition of tumor growth. During 22 days treatment, there was no significant body weight loss in all four groups (Figure 5(B)). Furthermore, the antitumor efficacy was also assessed by H&E staining of tumor tissues after 22 days of chemotherapy (Figure 5(C)). Compared with the PBS and Gd/CeO_2_-ZrO_2_-PEG treated groups, the cancer cells in the free DOX or Gd/CeO_2_-ZrO_2_/DOX-PEG treated groups showed a typical feature of cell necrosis with obvious cytoplasm leakage and nuclear shrinkage. However, compared with a portion of malignant cells maintaining normal state in group of free DOX, nearly all the cells underwent necrosis in group of Gd/CeO_2_-ZrO_2_/DOX-PEG. Simultaneously, the immunohistochemical (IHC) staining of tumor sections for antigen Ki67 was applied to analyzed cell proliferation after 22 days of chemotherapy treatment. In Figure 5(C), the cancer cells grew rapidly and cell mitosis could be clearly observed in the tumor tissues in the control group (PBS) and the Gd/CeO_2_-ZrO_2_-PEG treated group. In the group administrated with free DOX, cell proliferation was hindered and several areas of necrosis were seen. It is gratifying to be that a greater degree of tumor necrosis in the group treated with Gd/CeO_2_-ZrO_2_/DOX-PEG was observed, suggesting its superior efficiency in inhibiting proliferation and inducing apoptosis of tumor cells.

### 3.8 *In vivo* MR and CT imaging

Moreover, the *in vivo* tumor accumulation ability of Gd/CeO_2_-ZrO_2_/DOX-PEG nanoparticles was investigated on HepG-2 tumor-bearing nude mice by MR and CT imaging. Firstly, T_1_-weighted MR imaging capability of the prepared theranostic nanoplatform was studied by using a 7.0 T MRI scanner. As shown in Figure 5(D), the tumors on mice exhibited a trend of brightening effect over time with the administration of Gd/CeO_2_-ZrO_2_/DOX-PEG nanoparticles. After 4 hours of tail intravenous injection, the signal reached the strongest. The T_1_ imaging results were then quantitatively analyzed through calculating the region-of-interest (ROI) gray values. As shown in Figure S6A, the gray value of tumor site was increased from 144.3 (0 h post-injection) up to 317.2 (4 h post-injection). Overall, these results suggested that the Gd/CeO_2_-ZrO_2_/DOX-PEG could act as a potential MRI contrast enhancing agent for tumor imaging. Thereafter, we have further investigated the enrichment of nanoparticles in tumor tissue by CT imaging. As shown in Figure 5(E), the CT signal of tumor site increased gradually, and the signal displayed similarly the strongest after 4 h. As shown in Figure S6B, the CT signal intensity of tumor site was increased from 40.3 (0 h post-injection) up to 76.2 (4 h post-injection). In addition, the effects of clinically used contrast agents have been evaluated. Compared with 0 h post-injection, the MR and CT signals have no significant increase after 4 h of injection, which was due to the rapid clearance of these small molecules by reticuloendothelial system (Figure S7). These results indicated that our Gd/CeO_2_-ZrO_2_/DOX-PEG nanoparticles could continuously accumulate in tumor tissues as a result of passive EPR effects.

## 4. Conclusions

In this work, we have successfully prepared the Gd/CeO_2_-ZrO_2_/DOX-PEG nanoplatform as multifunctional cancer theranostic system possessing MR/CT dual-model imaging and chemotherapy ability. The obtained carrier not only exhibited well-defined shapes, tunable compositions and narrow size distributions, but also showed well biocompatibility *in vitro* or *in vivo*. The tumor growth was highly suppressed due to the efficient delivery of nanoplatform to tumors. Moreover, the nanoplatform could be an informative MRI and CT dual-model contrast agent for tumors. In other words, such a single nanoplatform with dual-model imaging would be of great value to offer more comprehensive diagnostic information and more accurate location. Therefore, the present Gd/CeO_2_-ZrO_2_/DOX-PEG nanoparticles can be served as a promising theranostic nanoplatform using dual MR/CT imaging and chemotherapy of tumor.

## Supplementary Material

IDRD_Yi_et_al_Supplemental_Content.doc
