# Supplementary material for: Gadolinium-doped hollow CeO2-ZrO2 nanoplatform as multifunctional MRI/CT dual-modal imaging agent and drug delivery vehicle
Source: Drug Deliv. 2018 Jan 25;25(1):353–63. doi: 10.1080/10717544.2018.1428241 (PMC6058605; doi:10.1080/10717544.2018.1428241)
Supplement: IDRD_Yi_et_al_Supplemental_Content.doc [file IDRD_A_1428241_SM4450.doc]

**Supplemental file**

**Gadolinium-doped hollow CeO_2_-ZrO_2_ nanoplatform as multifunctional MR/CT dual-modal imaging agent and drug delivery vehicle**

*Zuwu Wei ^b^, Ming Wu ^b^, Zuanfang Li ^d^, Zhan Lin ^b^, Jinhua Zeng ^a,b^,* *Haiyan Sun ^e^, Xiaolong Liu ^b^, Jingfeng Liu ^a,b^, Buhong Li ^c*^ and Yongyi Zeng ^a*^*


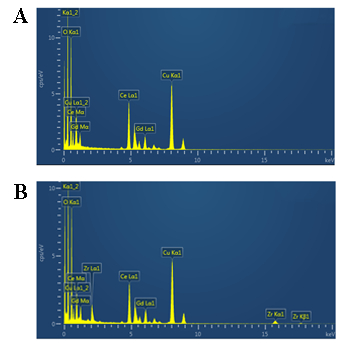


**Fig. S1.** Energy dispersive X-ray (EDX) spectrum of Gd/CeO_2_ (A) and Gd/CeO_2_-ZrO_2_.


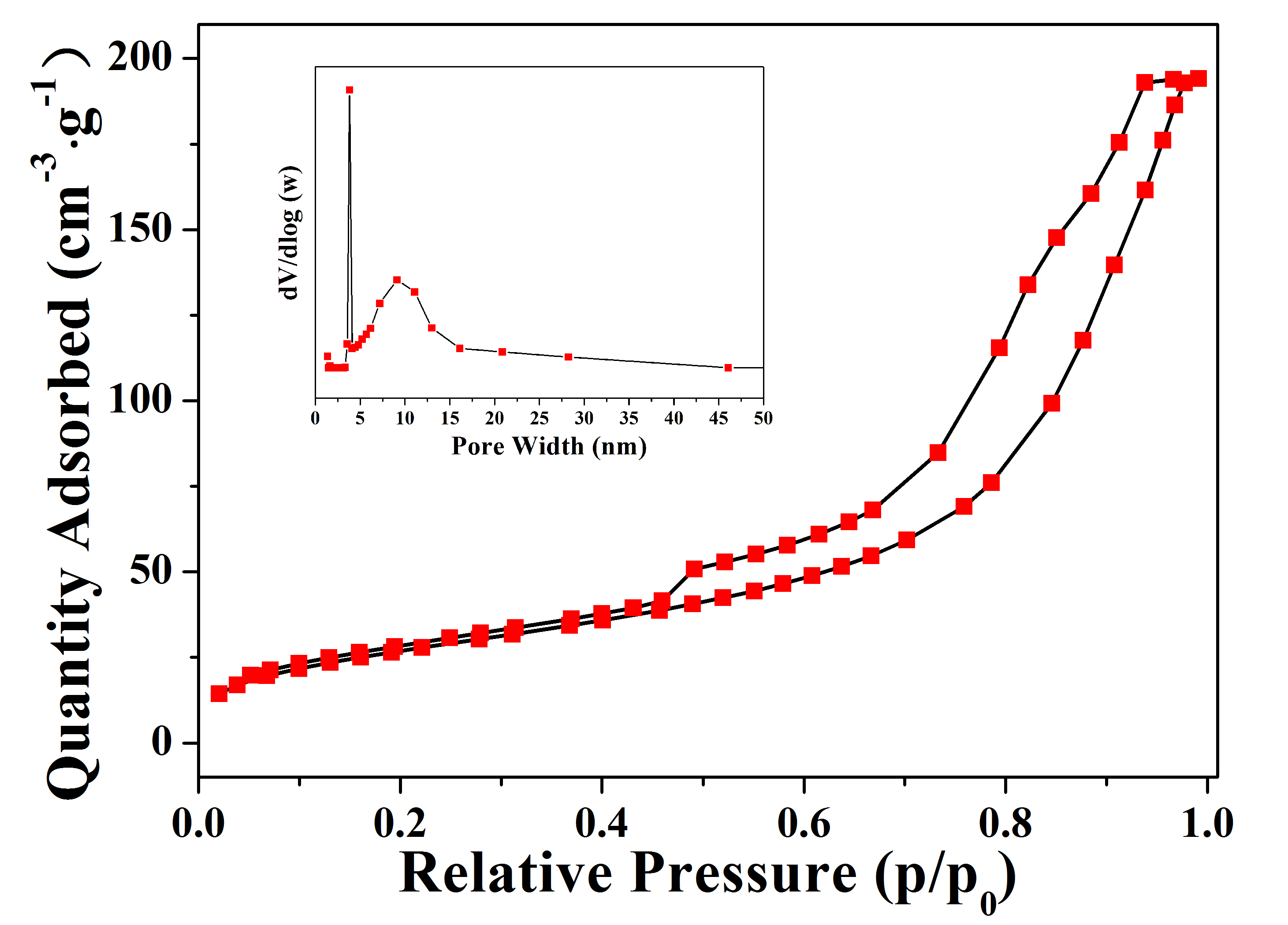


**Fig. S2.** N_2_ adsorption-desorption isotherms of Gd/CeO_2_-ZrO_2_-PEG (inset: the pore size distribution of Gd/CeO_2_-ZrO_2_-PEG).


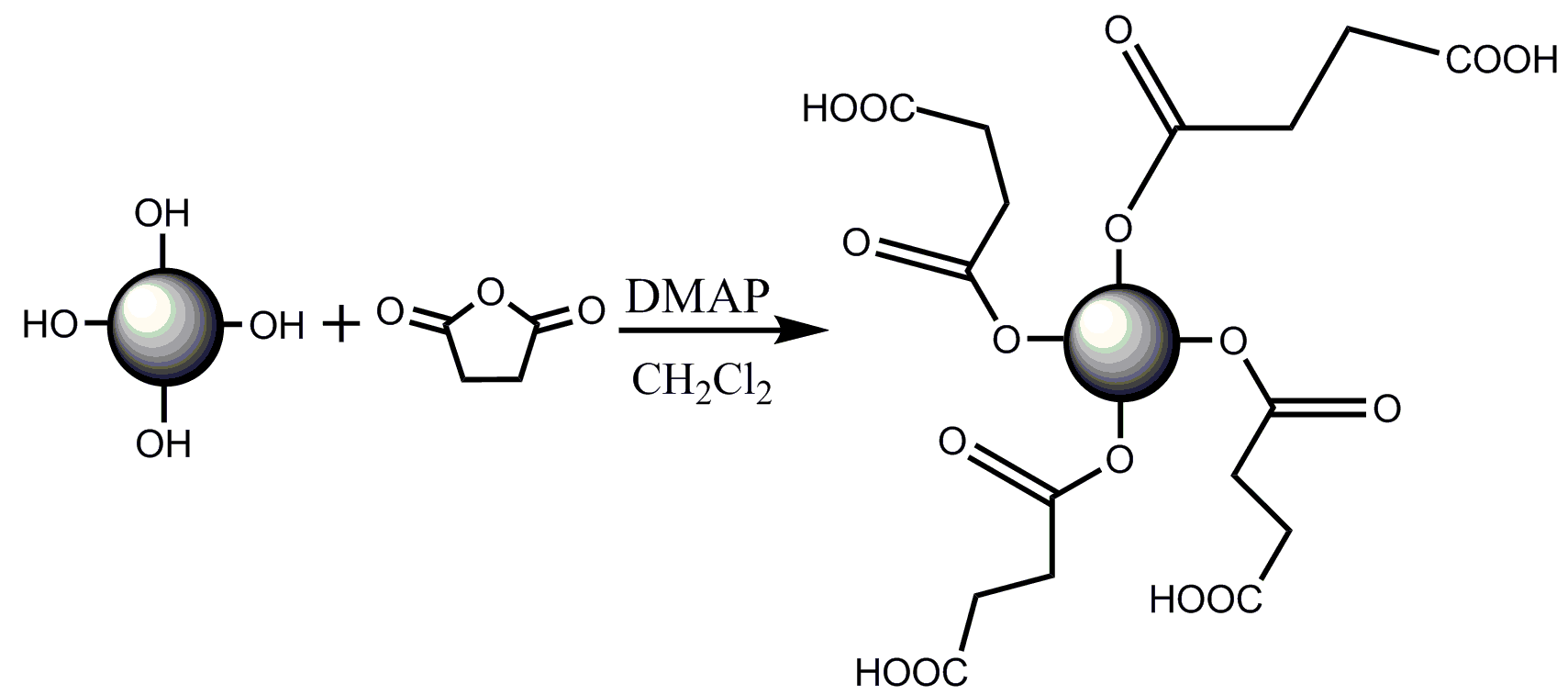


**Fig. S3.** The Gd/CeO_2_-ZrO_2_ reacted with succinic anhydride and generated Gd/CeO_2_-ZrO_2_-COOH.


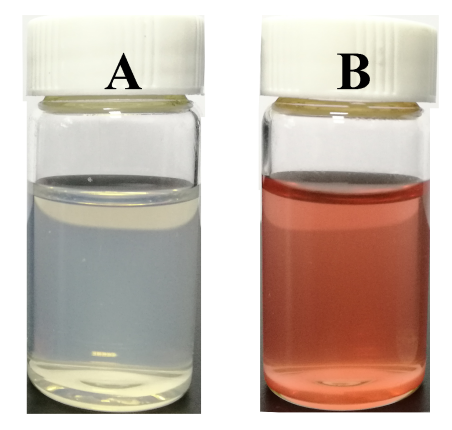


**Fig. S4.** Photograph of Gd/CeO_2_-ZrO_2_-PEG (A) and Gd/CeO_2_-ZrO_2_/DOX-PEG in PBS.


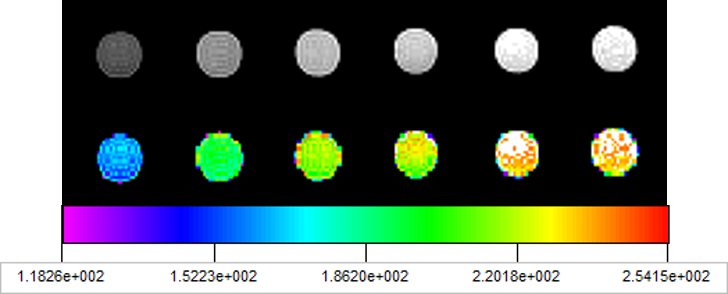


**Fig. S5.** CT images and color-mapped images for various Zr^4+^concentrations Gd/CeO_2_-ZrO_2_.

**
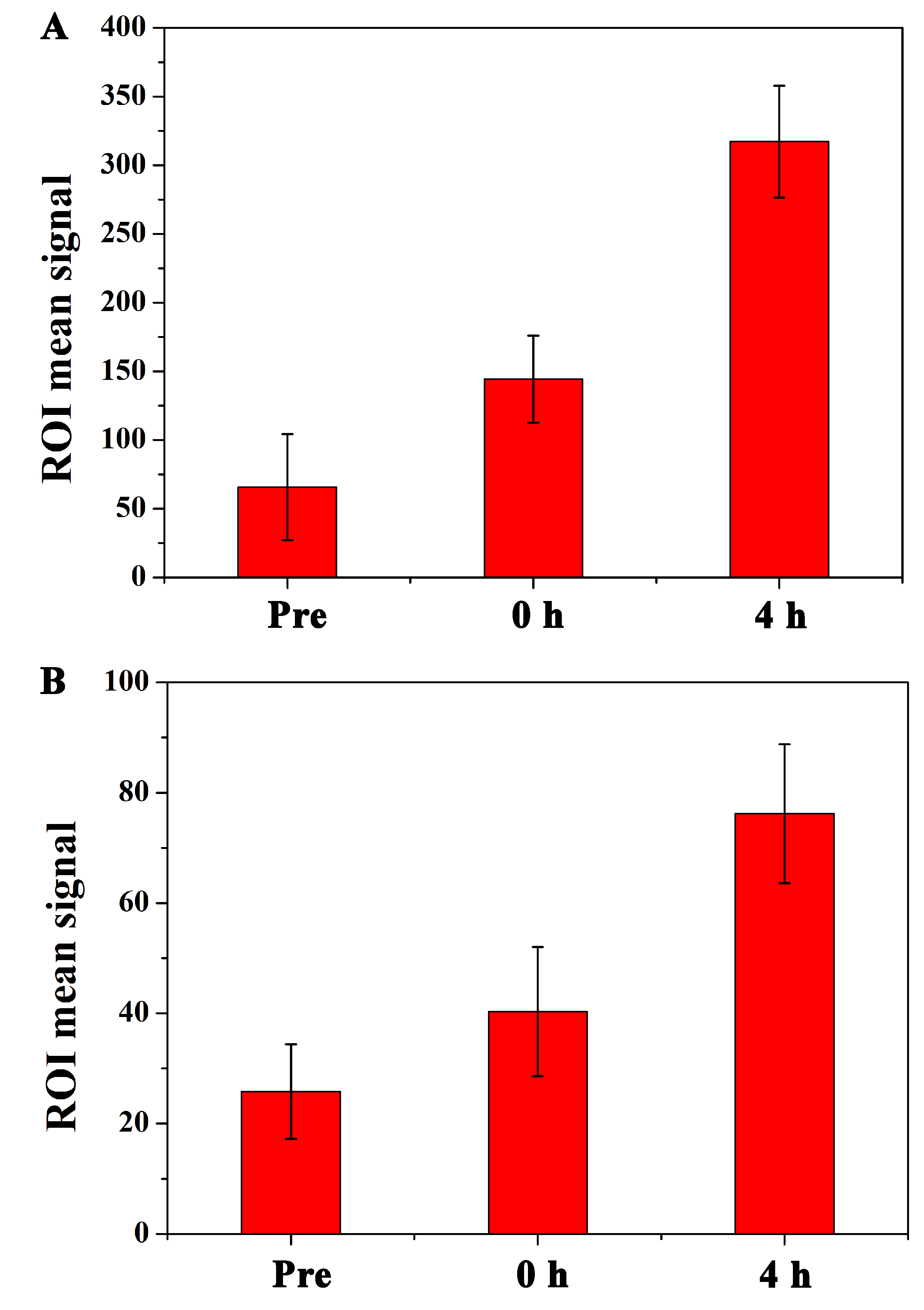
**

**Fig. S6.** The gray values of mice tumor, magnetic resonance images (A); computed tomography images (B).

**
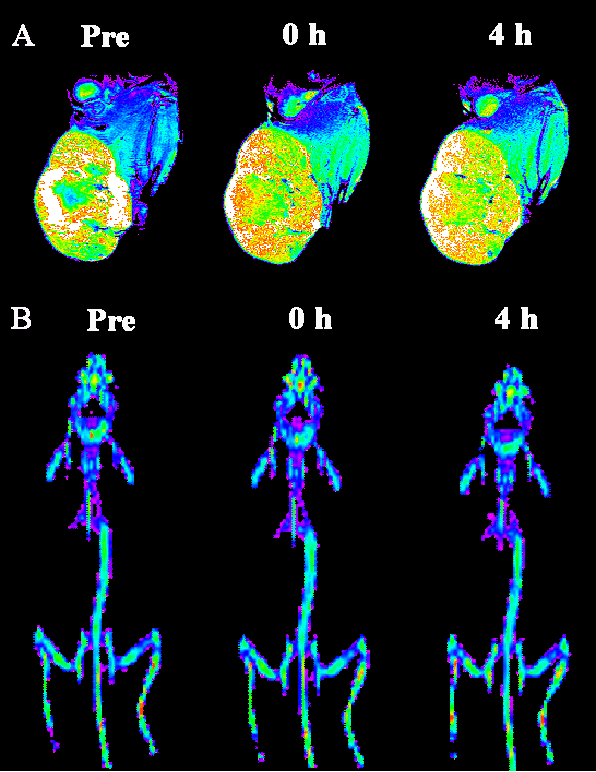
**

**Fig. S7** T_1_-weighted MR images of tumor acquired at different time intervals post injection of Gd-DOTA (D); CT images of tumor acquired at different time intervals post injection of Ioversol (E).
